# Editorial: Translating NK cell scientific research to clinical product manufacturing

**DOI:** 10.3389/fimmu.2023.1229417

**Published:** 2023-08-23

**Authors:** Evelyn Ullrich, Volker Huppert, Torsten Tonn, Wing Leung

**Affiliations:** ^1^ Experimental Immunology & Cell Therapy, Department of Pediatrics, Goethe University Frankfurt, Frankfurt, Germany; ^2^ Frankfurt Cancer Institute, Goethe University, Frankfurt am Main, Germany; ^3^ German Cancer Consortium (DKTK) and German Cancer Research Center (DKFZ) Partner Site Frankfurt/Mainz, Frankfurt am Main, Germany; ^4^ Glycostem Therapeutics B.V., Oss, Netherlands; ^5^ Transfusion Medicine, Medical Faculty Carl Gustav Carus, TU Dresden and Institute for Transfusion Medicine Dresden, German Red Cross Blood Donation Service North-East, Dresden, Germany; ^6^ Department of Pediatrics, The University of Hong Kong, Hong Kong, Hong Kong SAR, China

**Keywords:** Natural Killer cells, cell therapy, ATMP, good manufacturing practice (GMP), tumor therapy, manufacturing, NK cells

Natural Killer (NK) cells have a unique role of detecting and destroying malignant and virus infected cells (1) as a part of the immune system. The role of NK cells in the treatment of leukemia has been demonstrated in the context of hematopoietic stem cell transplantation (2, 3). These findings supported the use of allogeneic NK cells for treatment of patients and clinical responses by adoptive NK cell therapy have been observed [(4-7) reviewed in Lamers-Kok (8)].

Furthermore, during the last decade, genetic modifications of immune cells, namely T lymphocytes, to express chimeric antigen receptors have resulted in breakthrough clinical advancements in previously incurable B cell malignancies. Today, several CAR-T cell therapies have obtained marketing authorization and have become standard of care. In an effort to expand this clinical success achieved in liquid cancers to also solid cancers, NK cells have regained considerable interest as alternative “CAR-drivers”. NK cells have several attributes that make them an attractive effector cell for CAR-targeted cancer immunotherapies, especially when such treatments have to be made available to large cohorts suffering from more frequent malignancies, such as lung cancer, breast cancer and ovarian cancer. I) NK cells do not elicit GvHD reactions and are therefore a potential source for targeted - but with regard to the patient “undirected” - allogeneic off-the-shelf cell therapies; II) NK cells, in contrast to CAR-T cells, do not secrete IL-6, a hallmark cytokine causing severe complications described as cytokine release syndrome (CRS); III) NK cells may be able to activate the patient´s own immune system *via* dendritic and bystander T cells, which could potentially result in a long-lasting anti-tumor vaccine effect.

However, compared to CAR-T cells, CAR-NK cells are currently subjected to fewer clinical trials and the observed clinical benefits have been marginal. Nevertheless, the group around Winfried Wels from Frankfurt/Main, Germany, has established remarkable safety of intracranial injections of Her2 CAR-engineered NK-92 cells in patients with relapsed glioblastoma (9), and Rezvani and colleagues from MD Anderson, Houston, showed that systemic infusions of CD19 CAR engineered primary NK cells in patients with B cell malignancies resulted in signs of clinical benefit and NK cell persistence (10).

There are several shortfalls that can explain why NK cells did not quite keep up with the expectations yet, when it comes to CAR-effector cell based treatments. In contrast to T lymphocytes, NK cells are difficult to genetically engineer and, higher cell dosages have to be given initially, since NK cells do not clonally expand as extensively *in vivo*.

With this Research Topic all devoted to the translation of NK cell based therapies into the clinic and to address above mentioned shortcomings of NK cells, Frontiers in Immunology provides an up-to-date insight in NK manufacturing, genetic engineering and the latest clinical development.

In the article by Johnson et al., NK Cloudz - a dissolvable polymer-based microsphere platform - was developed as an alternative to a feeder cell approach to expand NK cells. The authors demonstrated that a combination of NK Cloudz, a G-Rex6M culture vessel, and GMP Human Platelet Lysate could expand NK cells by almost 400-fold in 10 days from a PBMC starting population. In the work of Oyer et al., they reported that PM21-particle expanded NK cells (PM21-NK cells) can be cryopreserved for off-the-shelf approaches without losing their cytotoxicity and effector functions *in vitro* and *in vivo* .

In the work by Dezfouli et al., Hsp70 peptide (TKD)/IL-2-stimulated NK cells and anti-Hsp70 CAR T cells demonstrated comparable anti-tumor effects against colorectal cancer cells, albeit with somewhat differing kinetics. Thus, TKD/IL-2-stimulated NK cells, as well as anti-Hsp70 CAR T cells, provide a promising direction to target mHsp70, which is frequently and specifically expressed on the cell surface of many different cancers other than colorectal cancers. In the study by Soldierer et al., further optimizations in CAR-NK manufacturing for off-the-shelf applications were presented, including different internal promotors for lentiviral CAR vectors, lentiviral pseudotypes, viral entry enhancers, and IL-15 signaling. Coupled with the natural cytotoxicity of NK cells and the lack of graft-versus-host disease potential, testing of these CAR-NK cells for adoptive immunotherapy is warranted.

In the Mini Review by Gurney et al., the complex interactions were reported that exist between feeder cells and both viral and emerging non-viral genome editing technologies in NK cell engineering. They focus on two established clinical-grade feeder systems: Epstein-Barr virus transformed lymphoblastoid cell lines and genetically engineered K562.mbIL21.4-1BBL feeder cells. In the review by Boyd-Gibbins et al., insights in NK cell manufacturing of induced pluripotent stem cells (iPSCs) and purified NK cell extracellular vesicles (NKEVs) are provided, including the discussion if NKEV reproduce key functions of their parent NK cells and can be developed into a standalone therapeutic with reduced immunogenicity compared to cell therapies. The authors discuss the role iPSC technology might play in both NK cell manufacturing and NKEV development. Ruppel et al. provided a technological overview on tailoring design and signaling for CAR-NK cells in cancer therapy. The authors explore different CAR formats and modifications to optimize NK cell-mediated signaling, and also challenges beyond NK cell engineering, including expansion and manufacturing. The article by Tarannum et al. presents an up-to-date review on innovative strategies to improve the clinical application of NK cell-based immunotherapy, addressing key challenges that need to be solved for effective translation of NK cell research into clinical applications, including *in vitro* expansion, *in vivo* persistence, infiltration to the tumor site, and prevention of exhaustion.

Finally, while prior studies of NK cell therapy generally had short follow-up time on patients with clinical response, the report by Parisi et al. reported definitive evidence that donor-derived alloreactive KIR-ligand-mismatched NK cells can result in durable control of acute myeloid leukemia for more than 10 years. In recipients with a dose of infused alloreactive NK cells >2 x10^5/kg, the 5-year disease-free survival beyond first morphological complete remission was >60%.

Collectively, these clinical data and the exciting technological advances described in this series of articles pave the way for next generation of GMP manufacturing and clinical applications.

## Author contributions

EU, WL, TT and VH edited the submissions to the Research Topic, summarized the conclusions and wrote the editorial. All authors contributed to the article and approved the submitted version.
